# Temporal evolution of vasospasm and clinical outcome after intra-arterial vasodilator therapy in patients with aneurysmal subarachnoid hemorrhage

**DOI:** 10.1371/journal.pone.0174676

**Published:** 2017-03-24

**Authors:** Laleh Daftari Besheli, Can Ozan Tan, Donnie L. Bell, Joshua A. Hirsch, Rajiv Gupta

**Affiliations:** 1 Department of Radiology, Massachusetts General Hospital Boston, MA, United States of America; 2 Harvard Medical School, Boston, MA, United States of America; 3 Cerebrovascular Research Laboratory, Department of Physical Medicine and Rehabilitation, Spaulding Rehabilitation Hospital, Boston, MA, United States of America; Emory University School of Medicine, UNITED STATES

## Abstract

Intra-arterial (IA) vasodilator therapy is one of the recommended treatments to minimize the impact of aneurysmal subarachnoid hemorrhage-induced cerebral vasospasm refractory to standard management. However, its usefulness and efficacy is not well established. We evaluated the effect IA vasodilator therapy on middle cerebral artery blood flow and on discharge outcome. We reviewed records for 115 adults admitted to Neurointensive Care Unit to test whether there was a difference in clinical outcome (discharge mRS) in those who received IA infusions. In a subset of 19 patients (33 vessels) treated using IA therapy, we tested whether therapy was effective in reversing the trends in blood flow. All measures of MCA blood flow increased from day -2 to -1 before infusion (maximum Peak Systolic Velocity (PSV) 232.2±9.4 to 262.4±12.5 cm/s [*p* = 0.02]; average PSV 202.1±8.5 to 229.9±10.9 [*p* = 0.02]; highest Mean Flow Velocity (MFV) 154.3±8.3 to 172.9±10.5 [*p* = 0.10]; average MFV 125.5±6.3 to 147.8±9.5 cm/s, [*p* = 0.02]) but not post-infusion (maximum PSV 261.2±14.6 cm/s [*p* = .89]; average PSV 223.4±11.4 [*p* = 0.56]; highest MFV 182.9±12.4 cm/s [*p* = 0.38]; average MFV 153.0±10.2 cm/s [*p* = 0.54]). After IA therapy, flow velocities were consistently reduced (day *X* infusion interaction *p*<0.01 for all measures). However, discharge mRS was higher in IA infusion group, even after adjusting for sex, age, and admission grades. Thus, while IA vasodilator therapy was effective in reversing the vasospasm-mediated deterioration in blood flow, clinical outcomes in the treated group were worse than the untreated group. There is need for a prospective randomized controlled trial to avoid potential confounding effect of selection bias.

## Introduction

Aneurysmal subarachnoid hemorrhage (aSAH) affects 21,000 to 33,000 individuals each year in the U.S., and accounts for approximately 5% of new stroke cases. It occurs at a young age, over 40% of the patients die within 28 days, and survivors often experience long-term cognitive and physical disabilities [1,2,3,4]. Thus, despite major advances in acute care, the prognosis for patients with aSAH remains poor.

Cerebral vasospasm and delayed cerebral ischemia (DCI), are the leading causes of mortality and morbidity after aSAH [4,5,6,7]. Symptomatic vasospasm and cerebral ischemic injury (i.e., DCI) together account for nearly 50% of the early deaths in those who survive the initial hemorrhage and aneurysm treatment [3,8]. Therefore, it is clear that any intervention to minimize the incidence and impact of cerebral vasospasm and DCI may improve clinical outcome.

Intra-arterial (IA) vasodilator therapy is one of the recommended treatments to minimize the impact of aSAH-induced symptomatic cerebral vasospasm refractory to standard medical management. However, its usefulness and efficacy is not well established (Class 2b, Level B evidence) [9]. Various IA vasodilators have been evaluated for the treatment of refractory vasospasm including papaverine, verapamil, nimodipine, milrinone and nicardipine [10,11,12]. Of these, nicardipine is a second-generation dihydropyridine-type calcium channel blocker that has vasodilator properties with a unique cerebrovascular profile. It is administered routinely via continuous intravenous [13] or IA infusion [14,15], or as prolonged-release implants [16,17,18]. However, as conceded in the current guidelines, the primary limitation of IA vasodilator therapy is its short duration of benefit [19].

While large case series have suggested angiographic and clinical improvement after IA vasodilator therapy, there have been no randomized clinical trials. In fact, a recent meta-analysis of available studies reported contradictory results regarding the efficacy of vasodilator therapy regardless of administration route [20], and one study reported no differences in 90-day outcomes between those treated with intraventricular calcium channel blockers and historical controls [21]. This may be because while there is a correlation between severity of vasospasm and DCI, the two are not always causally linked [22], and treatment of vasospasm may not always translate to favorable clinical outcomes. Therefore, although IA vasodilator therapy is considered part of standard care, its efficacy, vis-à-vis clinical outcomes, remains equivocal. In this retrospective pilot study of 115 patients, we sought to evaluate the efficacy of IA vasodilatory therapy (nicardipine with or without milrinone infusion) in reversing aSAH-induced vasospasm and improving discharge outcomes.

## Methods

We retrospectively obtained medical records for all patients aged 18 years or older who were admitted to our neurologic intensive care unit (NICU) from 2005 to 2013 with a diagnosis of aSAH ascertained by admission CT, MRI, or digital subtraction angiography. The exclusion criteria were recurrent aneurysmal rupture, mycotic aneurysms, rupture-related intraparenchymal hemorrhage, anticoagulation/antiplatelet use at presentation, procedure-related intraparenchymal hemorrhage, and/or incomplete medical record. We also excluded patients who were treated with Hyperglide balloon and NeuroFlo perfusion augmentation device. The study was approved by Massachusetts General Hospital Institutional Review Board (Protocol 2014P002368).

For each eligible patient, we obtained age, sex, length of NICU stay, Hunt & Hess grade [23], Fisher grade [24], number of IA vasospasm treatments, presence of ischemic stroke, Charlson comorbidity index [25], and hospital discharge modified Rankin Scale (mRS) Score (primary outcome measure). Patient records were de-identified and analyzed anonymously.

### Intra-arterial nicardipine therapy

While most high volume centers use balloon angioplasty for severe vasospasm, at our institution, pharmacologic treatment for vasospasm is preferred over mechanical interventions due to its safety profile and usefulness in treating the microvasculature [5], and endovascular treatment of vasospasm using IA nicardipine with or without milrinone is the standard of practice for patients who are refractory to optimal medical management. The refractory status is defined as the development of focal neurologic deficits attributable to cerebral vasculature with elevated TCD velocities and unexplained by established cerebral infarction (ascertained by perfusion imaging or MRI), hydrocephalus, recurrent aneurysmal hemorrhage or other medical comorbidity. Optimal medical management has historically been defined as medical therapy (including oral nimodipine) in addition to implementation of triple-H therapy.

IA therapy was administered between days 1 and 15 post-ictus when clinically indicated. Per routine practice in our institution, clinical indication for IA therapy was primarily based on neurological deterioration observed during patients’ clinical exams. For patients for whom a clinical exam was difficult to get, radiographic examination, typically transcranial Doppler ultrasonography, was relied upon to determine clinical indication. For IA vasodilator administration, the common femoral artery is accessed using a micropuncture kit. A 5 French sheath is placed and connected to a continuous flush. Subsequently, a 5 French Davis catheter is advanced over a J wire into the descending arch of the aorta and double flushed. The common carotid artery (CCA) and internal carotid artery (ICA) are then selectively catheterized using the Davis catheter and Terumo Glidewire. Biplane angiography is performed of these vessels. Next IA nicardipine is slowly administered, frequently followed by milrinone, while closely monitoring blood pressure and other vital signs. Angiography following injection is performed to confirm improvement. The diagnostic catheter, guide wire and sheath are then removed and hemostasis is achieved by manual compression and/or use of a closure device. Nicardipine and milrinone were administered, respectively, at 1 to 15 mg (average 7.6) and 5 to 15 mg (average 7.5) per vessel. The dosage was determined based on the observed satisfactory angiographic effect, the dose limit, or hemodynamic compromise.

### Transcranial doppler ultrasonography

In addition to discharge outcome, we also sought to establish the cerebrovascular hemodynamic effect of IA vasodilator therapy on cerebrovascular circulation. To this end, daily clinical transcranial Doppler Ultrasonography (TCD) values were obtained for the subgroup of patients who received IA vasodilator infusions. Clinical utility of TCD is established for detection and monitoring of angiographic vasospasm in patients with aSAH (Type A, Class I–II) [26]. In our institution, TCD measurements are performed daily or every other day beginning on day 1 post-ictus until discharge, for screening of vasospasm as part of the standard management. TCD is reliable for assessment of vasospasm in the middle cerebral artery (MCA), but its reliability decreases significantly for other brain vessels [9,26,27]. Hence, we only analyzed the TCD velocities at the MCA. We recorded maximum and average of the peak systolic velocities (PSV) and maximum and average of the mean flow velocities (MFV) across the cross-section of MCA. In case of unilateral IA vasodilator administration, we analyzed TCD measures obtained from the ipsilateral MCA.

### Statistics

A two-sample Wilcoxon Rank-Sum test was performed to compare Hunt & Hess grades, Fisher grades, Charlson comorbidity index and mRS in patients who received IA therapy and those who did not. We also performed an analysis of covariance for mRS controlling for sex, age, Charlson index, Hunt & Hess score, and Fisher grade.

In the subgroup of patients who received IA therapy, we assessed whether IA therapy was effective in reversing vasospasm. While an ideal control group would be a prospective cohort of patients who do not receive IA therapy despite medically refractory vasospasm, this is clearly not feasible as one cannot ethically withhold conventional treatment. Instead, because development of vasospasm and initiation of IA therapy varied relative to the onset of aSAH across patients, we first compared the differences in measures of MCA blood flow (average and maximum PSV and MFV) two days before (day -2), a day before (day -1), and a day after (day 1) each infusion. In this way, we were able to confirm the effectiveness of IA therapy independent of the postictal period duration. In addition, vasodilatory effects of IA nicardipine and milrinone can last 1–3 days. Therefore, for patients who received multiple infusions during the course of treatment (n = 5), subsequent injections were included in the analysis only if the interventions were at least three days apart, to eliminate potentially confound effect of prior administration. To account for multiple or bilateral (n = 10) infusions, we assessed the differences in measures of MCA blood flow via a linear mixed-effect model, repeated on each side (left/right, in case of bilateral infusions) and nested within each subject (for multiple infusions) with a random intercept.

Usually, vasospasm reaches a maximum and resolve over time. Therefore, it is possible that there would even be no significant difference in the MCA blood flow between days -1 and +1 without nicardipine administration. To avoid this confound, and to assess whether the effect of IA therapy lasted, one would ideally consider the time-course of MCA blood flow velocities before and after infusions in each case explicitly. However, this would require sufficiently and consistently long measurements for each patient, which was not practical given the retrospective nature of the current study. Instead, we relied on comparison of population-level trends in flow velocities before vs. after infusion. This was accomplished via an analysis of covariance (ANCOVA) to compare two regression lines (trend in blood flow velocities) by testing the effect of a categorical factor (before vs after IA vasodilator infusion) on the dependent variable (separately for each measure of MCA blood flow) while controlling for the effect of a continuous co-variable (day). This way, we tested the hypothesis that at the population level, the trend in blood flow velocities within days -8 to -1 (pre-infusion) would be different than that within days 1 to 10 (post-infusion), while also accounting for within-subject variability and correlations. A strong interaction between day and infusion would indicate an effect of IA therapy. Moreover, as a secondary confirmation, in a subset of individuals with sufficiently long data before and after infusion (n = 14 for maximum PSV, n = 8 for average PSV, and n = 5 for maximum and average MFV), we assessed the effect of infusion on an individual basis. Specifically, we tested the hypothesis that for each case, a change of trend in blood flow velocities will occur between days -1 and 1. To that end, for each individual, we fit a piecewise linear model with a single break-point with day (-8 to 10) as the independent variable, and measures of blood flow velocities separately as dependent variables. It is possible that changes in blood flow velocities due to vasospasm might not simply behave in a linear pattern. Nonetheless, we used a linear model given our limited number of observations and our aim to document the trend in blood flow, rather than accurate depiction of its behavior during vasospasm. If, for a given individual, the confidence interval of the estimated break-point intersected [–1, 1] interval, we classified that break-point as a “correct prediction.” This resulted in a dichotomous set of correct predictions (0 or 1). Subsequently, we assessed mean accuracy (and its 95% confidence intervals, given the small number of cases) for each measure of MCA blood flow velocity. In this way, we were able to reliably probe the effect of IA therapy despite our limited sample size.

Conformity of the data to assumptions that underlie statistical analysis was verified via standard tests, and data were transformed via Box-Cox transform if necessary. All analyses were performed using R Environment for Statistical Computing. *P* < 0.05 was considered statistically significant.

## Results

Of 115 patients who meet our inclusion criteria, 22 (19%) received IA therapy. Charlson comorbidity indices and Fisher grades at admission were not significantly different between patients who received IA therapy and those who did not. IA therapy group had significantly higher Hunt & Hess grade at admission (Table 1). mRS at discharge was higher in IA therapy group, even after adjusting for sex, age, Charlson index, Hunt & Hess, and Fisher grades. 7 out of 22 (32%) patients who received IA infusions had ischemic infarct during the course of hospital stay. In 3 of those, the infarct was present at admission, in 2 (9%), the infarcts were unlikely to be related to vasospasm, and in the remaining 2 (9%) the infarct could reliably be attributed to the vasospasm but not to the IA infusions (IA therapy was performed *after* the diagnosis of the infarct). None of the patients in IA therapy group died during the course of hospital stay. In contrast, 3/93 (3%) who did not receive any IA infusions died before discharge, although they did not have any vasospasm-related infarcts during their hospital stay. Length of ICU stay was significantly higher in IA therapy group. Clinical characteristics of these patients and the details of the IA therapy are shown, respectively, in Tables 2 and 3.

**Table 1:** Comparison between the Patient Groups.

	IA therapy (N = 22)	No IA Therapy(N = 93)	*P*
Age (Y)	52	54	0.4
Sex (% female)	77%	69%	0.4
Hunt & Hess grade (Mean; Median; IQR*)	3.1; 3; 2	2.3; 2; 2	0.004
Fisher grade (Mean; Median; IQR)	3.1; 3; 1	3; 3; 0	0.1
Charlson index (Mean)	0.5	0.6	0.3
Discharge mRS (Mean; Median; IQR)	2.6; 2; 2	1; 0; 1	<0.001
Length of NICU admission	18	12.2	<0.001

*Interquartile Range = Q3-Q1

**Table 2:** Clinical Characteristic of Patients Who Received IA Infusion.

Case #	Hunt and Hess	Fisher grade	Charlson comorbidity index	Co-morbidities	ICU stay (days)
2	4	3	0	No	20
17	3	3	0	No	18
18	4	4	0	No	18
20	2	4	0	No	15
23	3	3	7	HIV and mild liver disease	16
31	2	4	0	No	14
37	4	2	0	No	15
45	1	3	0	No	22
50	5	3	0	No	16
59	3	3	0	No	20
63	4	2	0	No	23
72	1	4	1	COPD	18
77	2	3	1	Asthma	21
79	4	4	0	N	38
85	4	4	0	No	18
92	3	3	0	No	17
95	3	2	1	Asthma	16
99	2	2	0	No	15
102	3	2	0	No	12
105	3	4	1	Diabetes Mellitus without end organ damage	15
111	5	3	0	No	18
114	4	4	1	Bronchitis	21

**Table 3:** Details of the IA Infusions.

Case #	Injected Vessel*	Nicardipine (mg)	Milrinone (mg)	Number of IA Treatments	Vasospasm-related infarct	Discharge mRS
2	R ICA	10	5	2	No	2
17	R ICA	5	-	1	No	2
18	R ICA	2	10	4	No	5
	L ICA	1	10			
20	R ICA	10	-	2	No	2
23	Inadequate TCD data			4	Yes**	4
31	L ICA	15	5	3	No	4
37	L ICA	10	-	2	No	1
	R CCT	10	5			
	L CCT	10	5			
45	R ICA	5	-	3	No	4
	L ICA	5	-			
50	L ICA	10	15	5	No	2
59	R ICA	5	5	1	No	3
	L ICA	5	-			
63	L ICA	4	5	1	No	3
72	R ICA	10	-	1	Yes**	4
	L ICA	10	-			
77	R ICA	2	5	4	No	2
	L ICA	4	5			
	R ICA	-	5			
	L ICA	5	10			
79	R ICA	10	15	10	No	2
	R ICA	15	15			
85	L ICA	5	5	1	No	3
92	L ICA	10	10	2	No	1
	R ICA	5	5			
	L ICA	10	5			
	R ICA	10	10			
95	Inadequate TCD data			5	No	0
99	L ICA	10	5	2	No	1
102	Inadequate TCD data			1	No	3
105	R ICA	7.5	-	1	No	1
	L ICA	5	-			
111	R ICA	10	5	1	No	4
114	R ICA	5	10	5	No	4
	L ICA	10	5			

*Only injected vessels that their baseline and follow-up TCD data were available were mentioned.

** IA therapy was performed after diagnosis of the infarct.

Of the 22 patients who received IA therapy, three did not have adequate baseline and follow-up TCD data. Thus, 19 patients with 33 treated vessels with baseline and follow-up TCD data were included in the subgroup analysis. IA nicardipine was used as monotherapy (i.e., without milrinone) in 10/33 (31%) injected vessels (Table 3).

**Fig 1:**
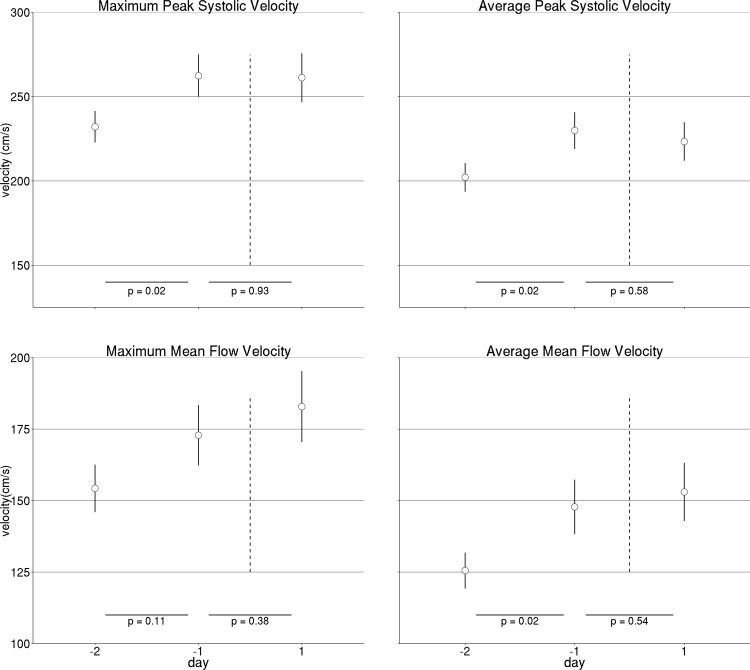
TCD Measures. TCD measures of middle cerebral arteries blood flow in 19 patients with aneurysmal subarachnoid hemorrhage who received IA vasodilator treatment (a total of 33 infusions) two days before (day -2), a day before (day -1), and a day after (day 1) infusion. Dashed vertical lines mark the infusion.

Consistent with vasospasm, all measures of MCA blood flow increased from day -2 to -1 before infusion: maximum PSV 232.2 ± 9.4 (mean ± SE) to 262.4 ± 12.5 cm/s (*p* = 0.02); average PSV 202.1 ± 8.5 to 229.9 ± 10.9 cm/s (*p* = 0.02); maximum MFV 154.3 ± 8.3 to 172.9 ± 10.5 cm/s (*p* = 0.10); average MFV 125.5 ± 6.3 to 147.8 ± 9.5 cm/s, (*p* = 0.02). In contrast, all measures of MCA blood flow were not different between days -1 (before infusion) and 1 (after infusion) (day 1 post-infusion maximum PSV 261.2 ± 14.6 cm/s (*p* = 0.89); average PSV 223.4 ± 11.4 cm/s (*p* = 0.56); maximum MFV 182.9 ± 12.4 cm/s (*p* = 0.38); average MFV 153.0 ± 10.2 cm/s (*p* = 0.54) compared to values at day -1 before infusion) (Fig 1). These results indicate that IA therapy mitigated progression of cerebral vasospasm.

**Fig 2:**
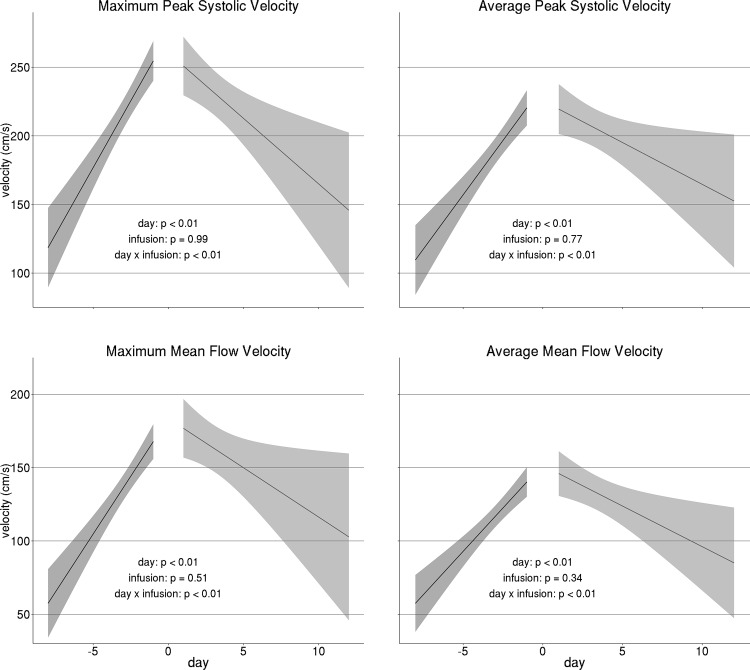
Trends in MCA Blood Flow. Trends in TCD measures of middle cerebral blood flow before and after intra-arterial infusion (day 0) across days. Shaded areas denote 95% confidence intervals. Strong day x infusion interaction indicates a significant reversal in trend after infusion.

At the population level, there was a clear reversal in the trend of MCA blood flow velocities after IA therapy. All measures of MCA blood flow velocity showed a trend towards increase across days before IA infusion, whereas they were consistently reduced across days after the treatment (day x infusion interaction *p* < 0.01 for all measures; Fig 2. Data is provided in S1 File). This result shows that IA therapy has a relatively lasting effect on reversal of vasospasm. In fact, based on this reversal of trend, we were able to predict the day of IA nicardipine infusion at an individual level (maximum PSV 11/14 correct predictions, 78% (confidence intervals: 49%– 95%) accuracy; average PSV 6/8 correct predictions, 75% (35%– 96%) accuracy; maximum MFV 4/5 correct predictions, 80% (28%– 99%) accuracy; average MFV 3/5 correct predictions, 60% (15%– 95%) accuracy).

## Discussion

Despite its apparent effectiveness in mitigating vasospasm and reversing the trend in MCA flow, our results do not support a significant benefit of IA vasodilator (nicardipine with or without milrinone) therapy as an effective approach for improving clinical outcomes.

Before IA therapy, there was a steady and significant increase in MCA blood flow velocities. This is consistent with a steady worsening of vasospasm, which precipitated IA intervention. After IA therapy, there was an alteration in this trend. Specifically, MCA blood flow velocities remained relatively unchanged, and even reduced over the next several days. This subgroup analysis is limited due to the small number of patients (19) who received IA therapy. Nonetheless, it is strongly supportive of the IA vasodilator treatment in halting and even reversing the vasospasm-mediated deterioration in blood flow following aSAH. This result is consistent with prior studies on the direct effects of IA vasodilator treatment on cerebrovascular hemodynamics. Tejada et al. evaluated angiographic appearance of arteries in 11 patients after IA nicardipine treatment, and have shown an increase of over 60% in the diameter of the treated vessels [15]. Nogueira et al. evaluated five patients with a SAH-induced vasospasm and showed that IA therapy improved mean transit time and cerebral blood flow in ischemic regions [28]. Badjatia et al. studied the effect of IA nicardipine treatment on cerebrovascular hemodynamics in 15 patients, and showed that treatment reduced blood flow velocities for up to four days without any sustained changes in intracranial pressure [14]. Thus, IA vasodilator therapy is clearly effective on reversing vasospasm.

However, despite the apparent effectiveness of IA therapy in resolving vasospasm, mRS at discharge was higher in our IA therapy group, even after adjusting for sex, age, and Charlson index, Hunt & Hess, and Fisher grades at admission. While at first surprising, this result is in fact consistent with other reports. For example, Tejada et al reported that despite the remarkable effect of IA nicardipine administration on artery diameter (above), the odds of a poor outcome after aSAH was not different between IA nicardipine treatment and control groups (0.13–2.46 95% CI) [15]. Similarly, Lu et al reported that while intraventricular nicardipine administration appears to be effective in treating vasospasm, there was no difference in clinical outcomes between nicardipine and control patients at 30 and 90 days [21]. Thus, it IA vasodilator therapy may be ineffective in preventing worsening of clinical outcomes. As mentioned in the introduction, delayed cerebral ischemia (DCI) is the primary culprit for morbidity and mortality after aSAH, and vasospasm and DCI are not the same. In fact, different studies (e.g. the CONSCIOUS-1 trail) showed a reduction of cerebral vasospasms did not influence clinical outcome [29,30]. In fact, DCI (and thus, morbidity and mortality) may involve different pathophysiological changes than vasospasm of the major cerebral arteries alone [31], and other mechanisms of ischemic injury not directly treated by IA therapy, such as cortical spreading depression, microthrombosis, and inflammatory responses, may play a major role [32,33]. It is possible that nicardipine might have a beneficial effect on microvasospasms. Future retrospective studies should integrate further imaging modalities (e.g. MRI- or CTP-based cerebral perfusion imaging or angiographic data.

Our study is not without its limitations. First, it is important to note that patients for whom IA therapy is not indicated constitute a biased control group. In other words, IA therapies are indicated only for those who are refractory to optimal medical management (as defined in the Introduction). Therefore, it is possible that the latter group is more prone to worse clinical outcomes compared to the former. The ideal control group should include patients for whom IA therapy is indicated but not administered; however, this is clearly unfeasible for obvious ethical reasons. It is also possible that the outcome of these critically ill patients may reflect a greater vulnerability of relatively minor in-patient complications, such as hypertension, or high risk inside-hospital transportation, such as from NICU to the angio suite. Second, some high volume centers regularly use balloon angioplasty, instead if IA therapy. Because IA therapy is the routine treatment for refractory vasospasm in our institution, and due to the retrospective nature of our study, we were unable to compare the efficacy of IA therapy to that of angioplasty. Lastly, a majority of our patients received IA nicardipine in combination with milrinone. This was unavoidable due to the retrospective nature of our study; the decision to whether or not administer milrinone was made based on clinical indication rather than research needs. Milrinone is a phosphodiesterase inhibitor with vasodilatory effects, and it has been previously shown to be effective in mitigating vasospasm. Therefore, our data cannot answer whether the effects of IA therapy (or its lack) can be attributed to nicardipine, milrinone, or both.

## Conclusions

The evidence from this pilot, retrospective cohort supports efficacy of IA vasodilator treatment in preventing, or even reversing the vasospasm after aSAH, but patients with an indication for IA vasodilator therapy still had a worse clinical outcome than patients treated with standard medical management. Thus, our results do not support a significant benefit of IA vasodilator therapy for improving clinical outcomes. Future prospective studies with larger cohorts are needed to compare the efficacy of IA therapy relative to alternatives (e.g., balloon angioplasty).

## Supporting information

S1 File Hemodynamic Data. Hemodynamic data set underlying the findings.
(XLSX)
